# Efficient chemo-enzymatic gluten detoxification: reducing toxic epitopes for celiac patients improving functional properties

**DOI:** 10.1038/srep18041

**Published:** 2015-12-22

**Authors:** Miguel Ribeiro, Fernando M. Nunes, Sofia Guedes, Pedro Domingues, Amélia M. Silva, Jose Maria Carrillo, Marta Rodriguez-Quijano, Gérard Branlard, Gilberto Igrejas

**Affiliations:** 1 CQ-VR, Chemistry Research Centre, Chemistry Department, University of Trás-os-Montes and Alto Douro, 5000-801 Vila Real, Portugal; 2 Department of Genetics and Biotechnology, University of Trás-os-Montes and Alto Douro, 5000-801 Vila Real, Portugal; 3 Functional Genomics and Proteomics Unity, University of Trás-os-Montes and Alto Douro, 5000-801 Vila Real, Portugal; 4 Chemistry Department, University of Aveiro, 3810-193 Aveiro, Portugal; 5 Centre for the Research and Technology of Agro-Environmental and Biological Sciences, University of Trás-os-Montes and Alto Douro (CITAB-UTAD), 5000-801 Vila-Real, Portugal; 6 Unidad de Genética y Mejora de plantas Departamento de Biotecnología, E.T.S. Ingenieros Agrónomos Universidad Politécnica de Madrid, 28040 Madrid, España; 7 Institut National de la Recherche Agronomique GDEC/UBP, UMR 1095, 63100 Clermont-Ferrand, France; 8 BIOSCOPE Group, UCIBIO-REQUIMTE, Chemistry Department, Faculty of Science and Technology, University NOVA of Lisbon, Caparica, Portugal

## Abstract

Protein engineering of gluten, the exogenous effector in celiac disease, seeking its detoxification by selective chemical modification of toxic epitopes is a very attractive strategy and promising technology when compared to pharmacological treatment or genetic engineering of wheat. Here we present a simple and efficient chemo-enzymatic methodology that decreases celiac disease toxic epitopes of gluten proteins improving its technological value through microbial transglutaminase-mediated transamidation of glutamine with *n*-butylamine under reducing conditions. First, we found that using low concentrations of amine-nucleophile under non-reducing conditions, the decrease in toxic epitopes is mainly due to transglutaminase-mediated cross-linking. Second, using high amine nucleophile concentrations protein cross-linking is substantially reduced. Third, reducing conditions increase 7-fold the transamidation reaction further decreasing toxic epitopes amount. Fourth, using *n*-butylamine improves gluten hydrophobicity that strengthens the gluten network. These results open the possibility of tailoring gluten for producing hypoallergenic flours while still taking advantage of the unique viscoelastic properties of gluten.

Celiac disease (CD) is a common immune-mediated enteropathy which occurs following exposure to gluten in genetically susceptible individuals. CD prevalence is approximately 1% within the U.S. and European populations and, to date, no pharmacological treatment is available and a strict, life-long gluten-free diet is the only safe and efficient treatment available^1^,^2^.

The high content of glutamine (30–35%) and proline (10–15%) residues in gluten proteins makes it resistant to complete proteolytic digestion, ensuring the survival of immunostimulatory epitopes to digestion^3^. A key step in the pathogenesis of CD occurs when certain gluten peptides, namely those derived from glutamine-rich gliadins, are deamidated by tissue tranglutaminase (TG2), increasing their affinity for HLA-DQ2 or HLA-DQ8. This generates a more vigorous CD4^+^ T-helper 1 T cell activation, which can result in intestinal mucosal inflammation, malabsorption, and numerous secondary symptoms and autoimmune diseases^2^,^4^.

Gluten-free products have been developed to meet dietary limitations associated with gluten intake disorders. Nevertheless there are serious technological challenges for developing these products due to the inability of gluten-free cereal flours to retain carbon dioxide in fermented products and in general these products present poor organoleptic and nutritional properties^5^.

Wheat is the only known cereal whose flours after mixing with water and kneading are able to form an unique viscoelastic dough due to the formation of a three dimensional network interconnected by disulphide bonds, the gluten^6^. The main protein components of gluten are the gliadins and glutenins that provide extensibility and elasticity to wheat dough, respectively^7^.

Based on the better understanding of the pathogenesis of CD, some gluten targeted non-dietary therapies have been developed as for example the use of genetic engineering to down-regulate gliadins gene expression or the hydrolysis of immunodominant gliadin peptides that resist intestinal proteases by exogenous endopeptidases, and others^2^.

In this sense, selective chemical modification of gluten in order to decrease the content of CD-toxic epitopes presents important advantages over the pharmacological treatment of the disease as the chemical modification of gluten seeks the elimination of the exogenous effector of the disease, or over the genetic engineering of wheat to down regulate gliadins, as it is now known that all gluten proteins and not only gliadins are toxic to at least some susceptible individuals^8^, and the chemical modification seeks to transform selectively the epitopes whatever the protein nature. Since the microbial transglutaminase (mTG) treatment of wheat proteins for production of hypoallergenic flour was documented^9^,^10^, other works were conducted using mTG and an amine nucleophile to detoxify wheat flour for celiac patients^11^,^12^,^13^,^14^,^15^. Detoxification was attributed to the selective modification of glutamine residues present in toxic epitopes by mTG (transamidation) using L-lysine, L-lysine methyl or ethyl esters that prevent the deamidation process carried out by TG2 present in the human body. Furthermore, mTG lacks deamidating activity^11^. All of these studies showed that the decrease of toxic epitope levels in gluten, either by modifying glutamine residues in toxic amino acid sequences, or hydrolysis of the digestion-resistant toxic peptides bearing toxic amino acid sequences, resulted in a decrease in toxicity of modified products for celiac patients by using cell lines^11^,^12^,^14^,^15^,^16^. Nevertheless, the use of mTG without further addition of amine nucleophile seems to have the same effect^9^,^10^. Also, the introduction of lysine residues or its ester derivatives in gluten proteins, the only tested amine nucleophiles in the mTG mediated gluten detoxification, changes gluten charge density certainly impacting negatively the rheological properties of gluten^17^,^18^.

Here, we report a novel strategy to efficiently modify substantial amounts of glutamine residues in gluten catalysed by mTG using *n*-butylamine as amine nucleophile and reducing conditions, resulting in a low level of CD toxic epitopes and reaction products without charge. By using high amine nucleophile concentrations, protein cross-linking is substantially reduced, promoting the transamidation of glutamine residues with *n*-butylamine. This reaction features mild conditions, amino acid specificity, scale-up potential and no inherent requirement for genetic engineering. The resulting modified gluten proteins, besides their low content in toxic epitopes, yield protein networks with higher resistance to extension than the unmodified proteins.

## Results

### Transamidation of wheat flour and gluten under non-reducing conditions

**Figure 1:**
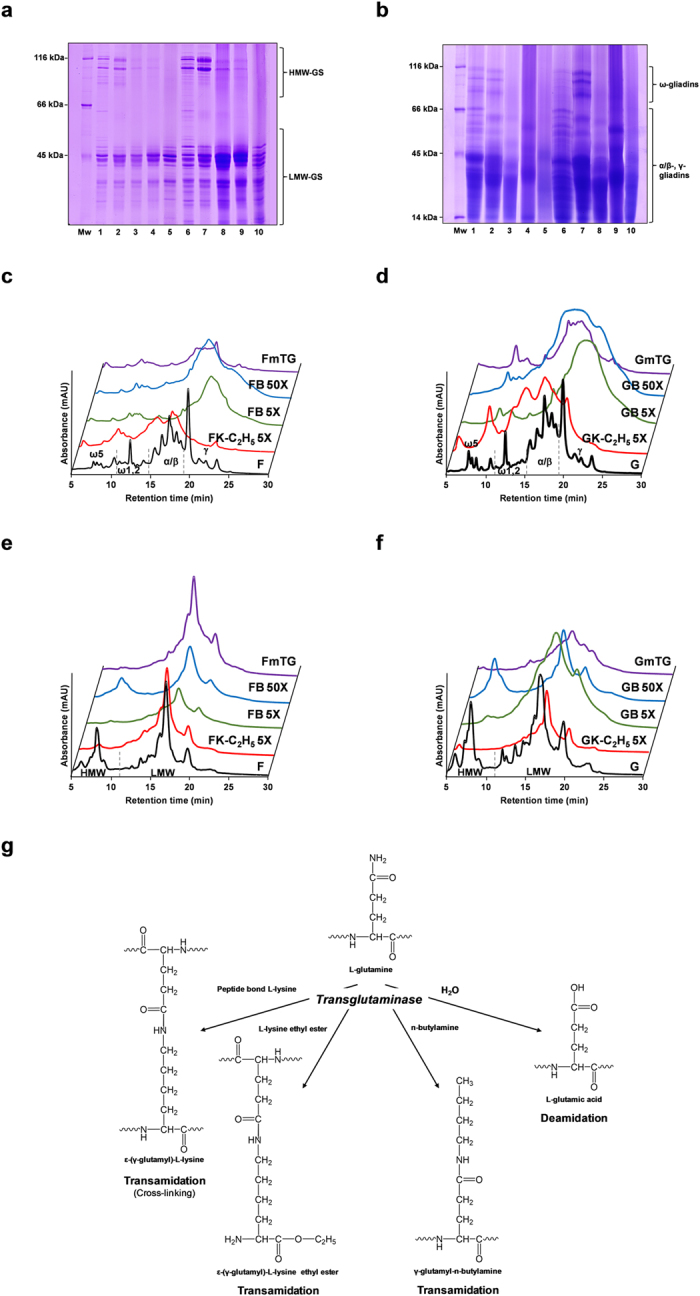
Structural changes in gluten proteins by transamidation of wheat flour and gluten under non-reducing conditions. (**a**) Reduced and alkylated glutenin subunit electrophoretic patterns of wheat flour and gluten, original and derivatised with mTG alone and with K-C_2_H_5_ and *n*-butylamine as amine nucleophiles under non-reducing conditions. (**b**) Gliadin electrophoretic patterns of wheat flour and gluten, original and derivatised with mTG alone and with K-C_2_H_5_ and *n*-butylamine as amine nucleophiles under non-reducing conditions. Lane 1, F; Lane 2, FB 50X; Lane 3, FB 5X; Lane 4, FK-C_2_H_5_ 5X; Lane 5, FmTG; Lane 6, G; Lane 7, GB 50X; Lane 8, GB 5X; Lane 9, GK-C_2_H_5_ 5X; Lane 10, GmTG. Reversed-phase HPLC results for gliadins (**c,d**) and glutenins (**e,f**) extracts of wheat flour and gluten, original and derivatised with mTG alone and with K-C_2_H_5_ and *n*-butylamine as amine nucleophiles under non-reducing conditions. Wheat flour chromatograms are represented for a maximum absorbance of 1.0 and gluten chromatograms are represented for a maximum absorbance of 2.5. ω5, ω1,2, α/β and γ represent the different identified gliadin proteins, and HMW and LMW represent the different glutenin subunits. Absorbance was registered at 210 nm. For sample nomenclature please consult Fig. 6. (g) Reactions catalysed by microbial transglutaminase and the end-products.

Wheat flour and gluten were allowed to react with *n*-butylamine in 5 and 50 molar excess in relation to the estimated glutamine residues^19^ in the presence of mTG (FB-5X, FB-50X, GB-5X and GB-50X, respectively). For comparison wheat flour and gluten were reacted with K-C_2_H_5_ in a 5 molar excess^11^,^12^,^13^,^14^,^15^ (FK-C_2_H_5_-5X and GK-C_2_H_5_-5X, respectively) and without any amine nucleophile in the presence of mTG (FmTG and GmTG, respectively). The same reaction conditions were used for wheat flour and gluten without mTG (F and G, respectively). The use of mTG without any amine nucleophile resulted in the disappearance of the high molecular weight-glutenin subunits (HMW-GS) in wheat flour and gluten (Fig. 1a), as previously described^20^, and also induced a smearing in the gel resultant from the presence of cross-linked proteins (Fig. 1g), although the low molecular weight-glutenin subunits (LMW-GS) were less modified.

**Figure 2:**
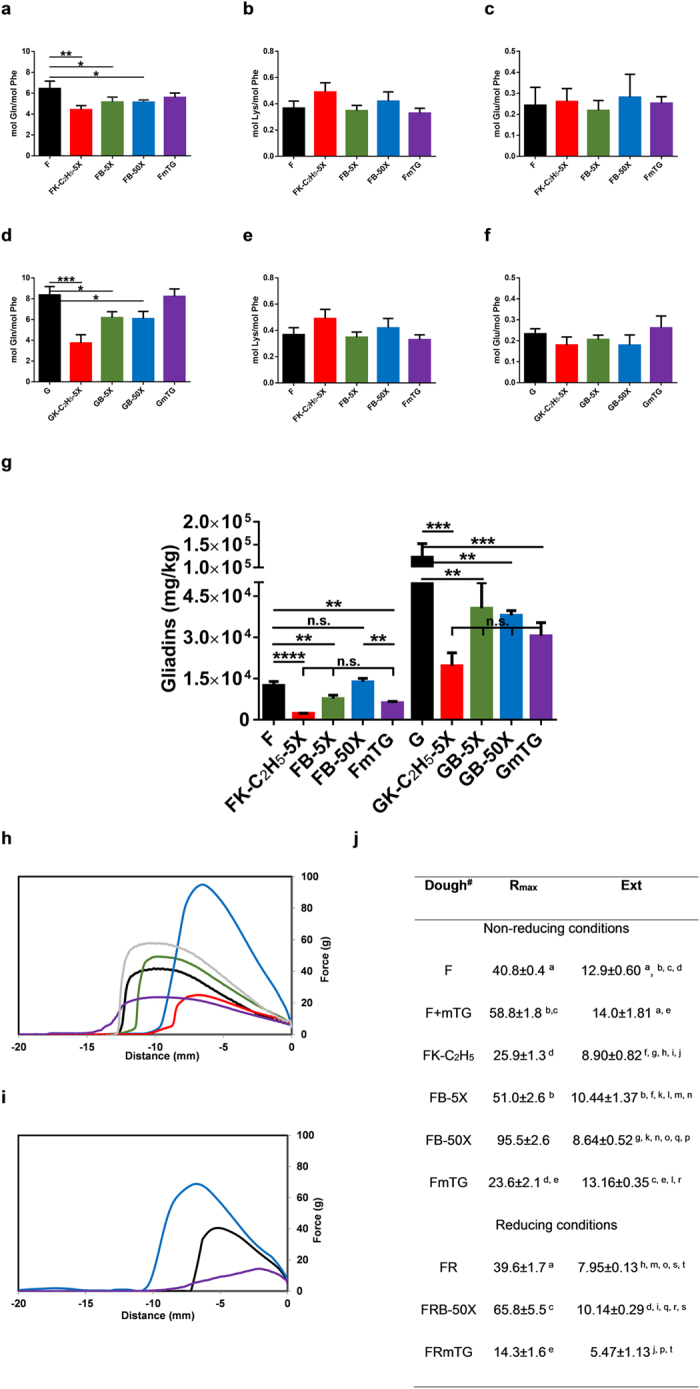
Cross-linking of gluten decreases toxic epitopes amount and affect negatively the rheological properties of gluten. Amino acid composition determined after enzymatic hydrolysis of wheat flour (**a**–**c**) and gluten (**d**–**f**), original and derivatised with mTG alone and with K-C_2_H_5_ and *n*-butylamine as amine nucleophiles under non-reducing conditions. For (**a**–**f**) error bars represent the standard deviation *p < 0.05, **p < 0.01, ***p < 0.001 and ****p < 0.0001 (n = 3). (**g**) R5 reactive epitopes’ content (mg of gliadin per kg of product) of wheat flour and gluten, original and derivatised with mTG alone and with K-C_2_H_5_ and *n*-butylamine as amine nucleophiles under non-reducing conditions after peptic-tryptic digestion. n = 2 different experiments (each with four replicates) and error bars represent the s.d. *p < 0.05, **p < 0.01, ***p < 0.001 and ****p < 0.0001. Micro-extension tests with dough prepared from wheat flour, original and derivatised with mTG alone and with K-C_2_H_5_ and *n*-butylamine as amine nucleophiles under non-reducing conditions. (**h**): 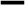 F; 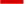 FK-C_2_H_5_; 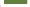 FB 5X; 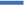 FB 50X; 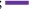 FmTG; 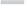 F + mTG. Or, under reducing conditions. (**i**): 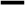 FR; 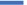 FRB 50X; 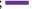 FRmTG. (**j**) Rheological properties of dough prepared from wheat flour, original and derivatised with mTG alone and with K-C_2_H_5_ and *n*-butylamine as amine nucleophiles under non-reducing and reducing conditions. ^#^For sample nomenclature please consult Fig. 6. The columns values with the same letter are not statistically significant, *p* < 0.05 (n = 2).

**Figure 3:**
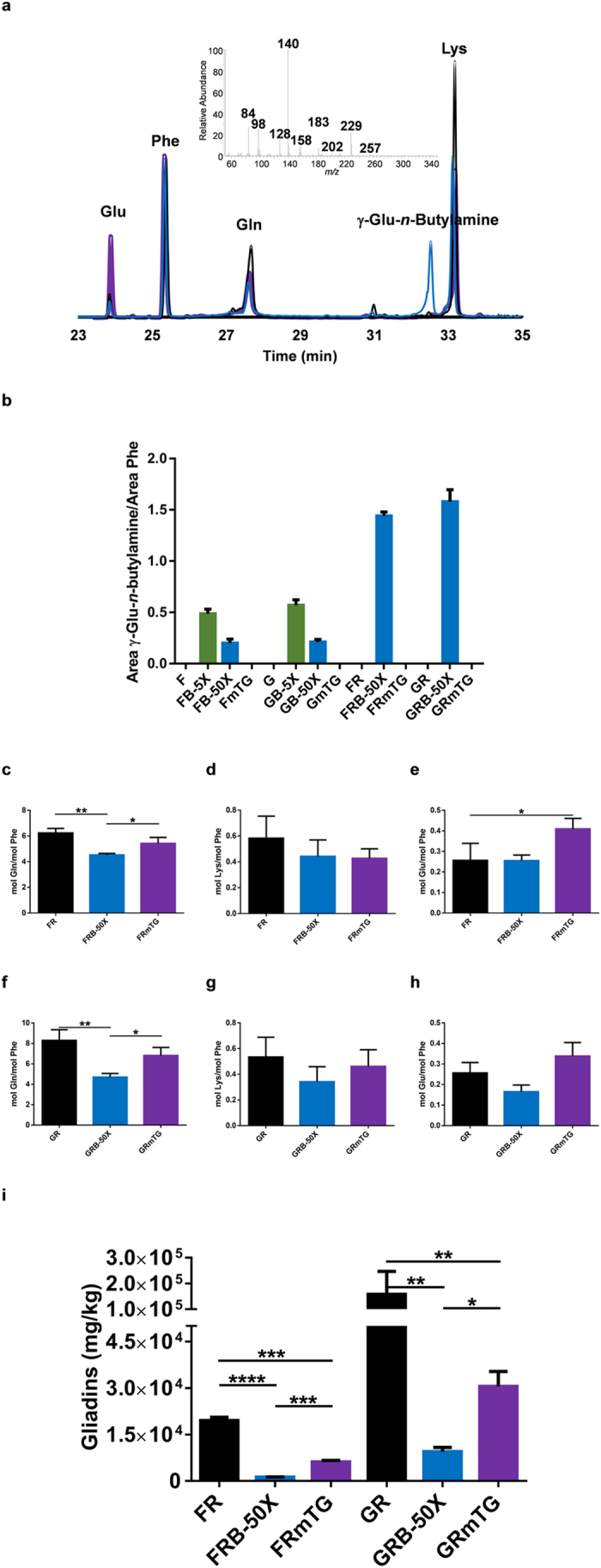
Reducing conditions favour the transamidation of gluten proteins and decreases further the amount of toxic epitopes. (**a**) GC-MS chromatogram for the amino acid analysis of 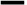 FR, 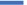 FRB 50X and 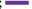 FRmTG. Indicated are the peaks of glutamic acid (Glu), phenylalanine (Phe), glutamine (Gln), γ-glutamyl-*n*-butylamine (γ-Glu-*n*-butylamine) and lysine (Lys). EI mass spectra of γ-Glu-*n*-butylamine residue is shown. (**b**) γ-Glu-*n*-butylamine residue formation under non-reducing and reducing conditions. For **b**, experiments were run in triplicate, and error bars represent the s.d. Amino acid composition determined after enzymatic hydrolysis of wheat flour (**c–e**) and gluten (**f–h**), original and derivatised with mTG alone and with *n*-butylamine as amine nucleophile under reducing conditions. For (**c–h**), error bars represent the standard deviation *p < 0.05, **p < 0.01, ***p < 0.001 and ****p < 0.0001 (n = 3). (**i**) R5 reactive epitopes’ content (mg of gliadin per kg of product) of wheat flour and gluten, original and derivatised with mTG alone and with *n*-butylamine as amine nucleophile under reducing conditions after peptic-tryptic digestion. n = 2 different experiments (each with four replicates) and error bars represent the s.d. *p < 0.05, **p < 0.01, ***p < 0.001 and ****p < 0.0001. For sample nomenclature please consult Fig. 6.

No distinct gliadin bands could be observed in wheat flour and gluten when treated with mTG alone (Fig. 1b), being only observed a smearing in the molecular weight range of the gels. Treatment of wheat flour and gluten with K-C_2_H_5_ and *n*-butylamine, in 5-fold molar excess, resulted in similar electrophoretic pattern for glutenins, with the bands of HMW-GS almost disappearing (Fig. 1a) and the loss of ω-gliadins and an intense smear of the α-, β- and γ-gliadins (Fig. 1b). For the wheat flour and gluten treated with 50-fold molar excess of *n*-butylamine the changes observed were much lower with an electrophoretic pattern for the glutenins and gliadins much more similar to the original wheat flour and gluten, being observed the HMW-glutenins (Fig. 1a) and also with distinct bands of ω-gliadins and α-, β- and γ-gliadins (Fig. 1b). Gliadin fractions presented more substantial change in the RP-HPLC profile (Fig. 1c,d) than the glutenin fraction, although some differences could also be observed in this fraction, mainly in the HMW-GS, being in accordance with the SDS-PAGE results (Fig. 1a). For the wheat flour and gluten modified with K-C_2_H_5_, proteins extracted in the gliadin fraction showed a decrease in their retention time, being in accordance with a decrease in hydrophobicity^21^ due to the introduction of positive charges resulting from the modification of glutamine residues with K-C_2_H_5_ (Fig. 1c,d). Contrarily, gliadins modified with *n*-butylamine increased their retention time, showing higher hydrophobicity (Fig. 1c,d). The higher susceptibility of gliadins to the chemical modification by the action of mTG is certainly related with their high content of glutamine residues^22^ and also due to the fact they are present essentially as monomeric proteins^23^. mTG treated flour and gluten also presented a modification in the RP-HPLC profile of gliadins, but the modifications observed are substantially different from that observed when flour and gluten were treated in the presence of K-C_2_H_5_ and *n*-butylamine, showing that the modifications observed by the action of mTG in the presence of the amine nucleophile are not entirely due to the cross-linking action of mTG (Fig. 1g). The lower extension of modification of the glutenins RP-HPLC profile and also retention time (Fig. 1e,f), even in the presence of mTG alone indicates that glutenins are more resistant to modification probably because in wheat flours, glutenins are present in macropolymers maintained by disulphide bonds^23^. In order to understand the changes in the chemical composition of the wheat flour and gluten proteins, and exclude deamidation of glutamine residues due to the action of mTG (Fig. 1g)^11^,^24^ that would be deleterious for the celiac disease related toxicity^1^,^4^, the amino acid composition of the wheat flours and glutens was determined. Under the assay conditions, no significant decrease in the lysine or increase in the glutamic acid levels of wheat flour and gluten proteins was observed (Fig. 2b,e,c,f). For the glutamine levels present in the wheat flour and gluten (Fig. 2a,d) treated with K-C_2_H_5_ it was observed a significant decrease (31% and 55%, respectively) in line with the higher substrate specificity of mTG for esterified L-lysine^25^,^26^. Also for the glutamine levels of wheat flour and gluten treated with a 5 and 50-fold excess of *n*-butylamine a significant decrease was observed (on average 20% and 27%, respectively). No significant decrease in the glutamine levels was observed for the wheat flours and gluten treated only with mTG (Fig. 2a,d). The amount of γ-glutamyl-*n*-butylamine resultant from the use of 50 fold excess of *n-*butylamine, either on the flour and gluten, was significantly lower (in average 60%) than that obtained when using 5 fold excess of *n-*butylamine (Fig. 3b). This result can be explained by the high concentration of *n*-butylamine in FB-50X and GB-50X reaction medium that inhibit the catalytic action of mTG as observed for human and guinea pig transglutaminases^27^ where it was observed that transglutaminase is able to bind the amine nucleophile in a manner that is not catalytically productive. This also can explain the lower extent of cross-linking observed for gliadins and HMW-GS when *n*-butylamine is used in 50-fold excess. It has been shown that mTG specificity is dependent not only on the primary sequence surrounding the reactive glutamine residue^26^,^28^, but also on the local secondary structure^29^, being postulated that local unfolding and peptide chain flexibility improve reactivity^30^. Under non-reducing conditions, the lack of reactivity of the remaining glutamine residues is likely related to the structural features of their local protein environment. This also explain the lower decrease in the amount of glutamine resides in FmTG and GmTG, where transamidation of glutamine residues only occurs with lysine residues present in some proteins from the reaction mixture, and after cross-linking, other reactive glutamine residues in the neighbourhood will be most probably inaccessible to mTG.

### Protein cross-linking decreases the amount of toxic epitopes

For accessing the potential toxicity of the final products for CD patients, the amount of toxic epitopes present in the samples was measured using the Codex Alimentarius standard and AACCI method (38–55.01), the R5 monoclonal antibody, that is known to recognize the potential celiac-toxic repetitive pentapeptide epitopes in gluten proteins^31^. Moreover, important and immunodominant stimulatory sequences are monitored by this method as for example the 33-mer, an α-gliadin derived peptide that resists to gastric/pancreatic digestion, ensuring the survival and maintenance of toxic epitopes, the 26-mer, a γ-gliadin derived peptide^3^, and others^32^. In order to avoid potential artefacts due to the possibility of changing the solubility properties of the gliadins and glutenins, either due to the structural changes imposed when modified with the amine nucleophile or due to cross-linking of proteins, the flours and glutens were submitted to a peptic-tryptic digestion process in order to release small peptides soluble in 60% (v/v) ethanol.

Treatment of wheat flour with mTG without amine nucleophile under non-reducing conditions resulted in a significant decrease, on average 50%, in the R5-reactivity (Fig. 2g). This reduction is comparable to the previously reported reduction of allergenicity of wheat proteins treated with mTG^9^,^10^ and the decrease in interferon (IFN) type II (also designed as IFN-γ) production of intestinal T-cells from celiac patients when exposed to mTG treated wheat flour’ peptides comparing to unmodified flour^11^. Also the treatment of wheat flour with mTG + K-C_2_H_5_ and mTG+ *n*-butylamine in 5-fold excess resulted in a significant decrease in toxic epitopes in relation to the unmodified flour, nevertheless the decrease observed (on average 81% and 38% in relation to unmodified flour, respectively) were not significantly different from that observed for the treatment with mTG alone (Fig. 2g). The same results were obtained when gluten was treated in the same conditions (Fig. 2g). For the FB-50X, on the contrary there was no significant decrease in the toxic epitopes in relation to the unmodified wheat flour and the levels were higher than those observed for the FmTG, FK-C_2_H_5_-5X and FB-5X, although the decrease of the content of glutamine residues in the FB-50X was similar to that found for FB-5X (Fig. 2a). These results indicate that, although glutamine residue levels in the flour were lower in the FK-C_2_H_5_-5X, FB-5X and FB-50X in comparison to unmodified flour and FmTG (Fig. 2a), only in the case of FB-50X the decrease in glutamine residue levels was not enough for reducing the R5-reactivity (Fig. 2g). On the other hand, for the GB-50X a significant reduction of the R5-reactivity was showed, although as observed in the electrophoretic profile (Fig. 1a,b), cross-linking of the gluten proteins was not extensive, as described also for the treated flour. This difference observed between the treatment of flour and gluten when using 50-fold excess of *n*-butylamine was attributed to the presence of albumins and globulins in the flour, which can account for approximately 22% of the total protein content in the flour, that are absent in the gluten^33^. As albumins and globulins are mainly monomeric and soluble in the reaction mixture they might be more accessible to the mTG and account for many of the reduction observed in the glutamine levels not resulting in a decrease in R5 reactivity, and in gluten the decrease in glutamine levels is only due to the modification of gliadins and glutenins. In addition, molecules of albumin and globulin which have higher lysine content can participate in cross-linking with glutenin^34^,^35^. The easiest modification of albumins and globulins explains the decrease in glutamine levels in all flours including FB-50X, but the much lower cross-linking observed for FB-50X results in the low detoxification of this flour. These results indicate that the cross-linking observed for the gluten proteins treated with mTG is the major factor contributing to the low reactivity observed for the FK-C_2_H_5_-5X and FB-5X (Fig. 1a,b).

### Rheological properties of dough are negatively affected by previous gluten cross-linking

Total extensibility (Ext) and maximum resistance to extension (R_max_) were measured using the Kiefer dough and gluten extensibility rig (Fig. 2h–j). The action of mTG on the wheat flour suspension resulted in a dough with a lower resistance to extension although not changing the extensibility when compared to the unmodified flour. This decrease in resistance to extension is due to the previous cross-linking of gluten proteins, especially the HMW-GS that are essential for the gluten network formation and the major determinants of the gluten strength^7^,^36^, that hamper a correct formation of the gluten network. This hypothesis is confirmed by the fact that when mTG is added to the unmodified flour (F+mTG; Fig. 2h,j) during the kneading process for dough formation the same results as those described in the literature are observed^34^, there is a significant increase of the maximum resistance to extension, although the extensibility was not significantly reduced. The derivatization of flour with K-C_2_H_5_ also resulted in a significant decrease in the R_max_ and also in Ext that can be due to either the cross-linking of the gluten proteins made by mTG, as described above, and/or due to the transamidation of the gluten proteins with K-C_2_H_5_ that results in the introduction of additional positive charges in the proteins due to the presence of the free α-amino group of K-C_2_H_5_. The increase in gluten protein charge density decreases protein interaction during gluten development by electrostatic repulsion^17^. On the other hand, the use of a 5-fold excess of *n*-butylamine resulted in dough with a significantly higher R_max_ than the original flour and the K-C_2_H_5_ derivatised flour. Also the treatment of flour with a 50-fold excess of *n*-butylamine resulted in dough with increased R_max_, which is significantly higher than the unmodified flour and FB-5X. The increase in R_max_, in this treatment, is explained by the fact that the previous mTG-induced cross-linking of gluten proteins is clearly lower than that observed for the FmTG, FK-C_2_H_5_-5X and FB-5X and also by the higher hydrophobicity of the gliadins in this treated flour that can reinforce the gluten network^37^. This reinforcement due to the increase in hydrophobicity of gliadins can also explain the higher R_max_ of FB-5X when compared to FmTG and FK-C_2_H_5_-5X. The lower value of R_max_ observed for FB-5X when compared to FB-50X, is explained by the higher previous mTG-mediated cross-linking in FB-5X when compared to FB-50X.

### Reducing conditions improve 7-fold the chemo-enzymatic modification of gluten proteins

**Figure 4:**
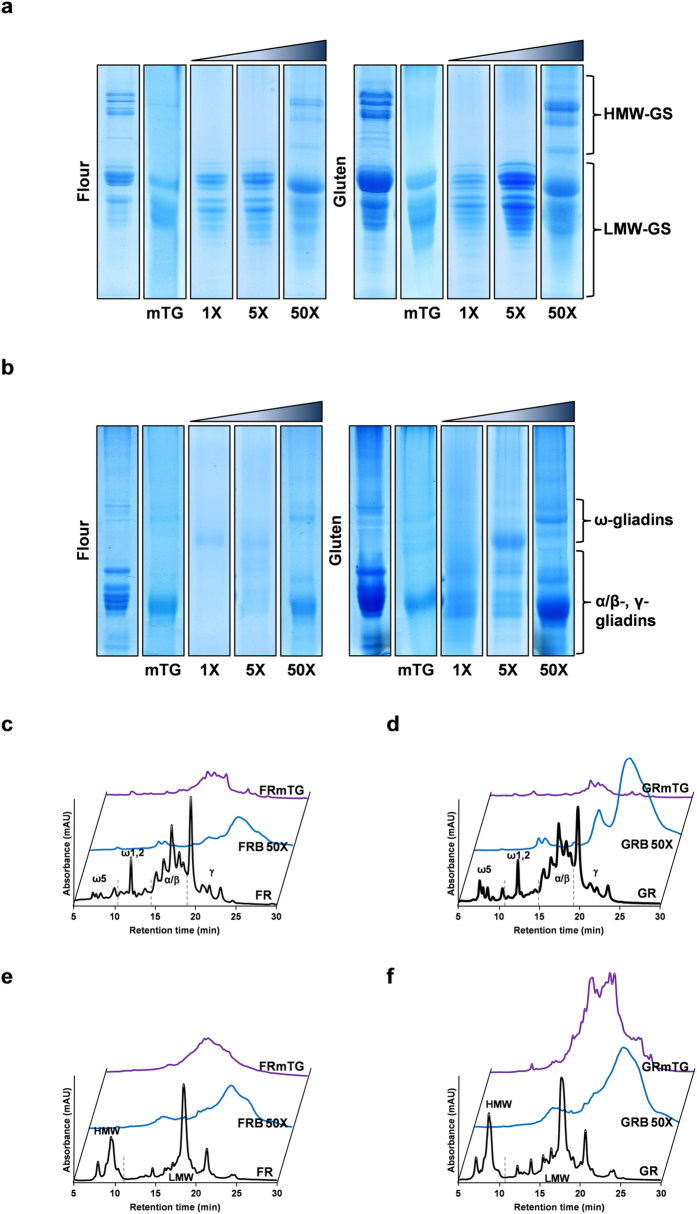
Structural changes in gluten proteins by transamidation of wheat flour and gluten under reducing conditions. (**a**) Reduced and alkylated glutenin subunit electrophoretic patterns of wheat flour and gluten (as denoted), original and derivatised with mTG alone and with *n*-butylamine as amine nucleophile with increasing concentrations under reducing conditions. (**b**) Gliadin electrophoretic patterns of wheat flour and gluten (as denoted), original and derivatised with mTG alone and with *n*-butylamine as amine nucleophile with increasing concentrations under reducing conditions. Reversed-phase HPLC results for gliadins (**c,d**) and glutenins (**e,f**) extracts of wheat flour and gluten, original and derivatised with mTG alone and with *n*-butylamine as amine nucleophile under reducing conditions. Wheat flour chromatograms are represented for a maximum absorbance of 1.0 and gluten chromatograms are represented for a maximum absorbance of 2.5. ω5, ω1, 2, α/β and γ represent the different identified gliadin proteins, and HMW and LMW represent the different glutenin subunits. Absorbance was registered at 210 nm. For sample nomenclature please consult Fig. 6.

We hypothesized that the observed limitation of mTG in decreasing the amount of CD toxic epitopes of gluten in the presence of excess of amine nucleophiles like K-C_2_H_5_ and *n*-butylamine when compared to the action of mTG alone is probably related to primary and secondary structure of gliadins and glutenins, besides the quaternary structure of glutenins, that can affect the glutamine accessibility to mTG and hamper their further chemical modification. Noteworthy is the observed resistance of LMW-GS to the transamidation by mTG under non-reducing conditions (Fig. 1a) as the abundance of glutamine residues in LMW-GS is comparable to that of the more mTG-susceptible gliadins^23^. A more extensive modification of all gluten proteins would reduce the amount of CD toxic epitopes from gluten proteins, and not only α/β-gliadins, as several studies have indicated that sulphur-rich prolamins, including α/β-gliadins and γ-gliadins, but also LMW-GS are the most active in celiac disease^8^,^38^. Gliadins are present essentially as monomeric proteins, with α/β and γ-gliadins with a conformation imposed by intramolecular disulphide bonds^23^ and glutenins are present in a polymeric structure (glutenin macropolymer), composed of HMW-GS and LMW-GS maintained by intermolecular disulphide bonds^23^ with molecular mass that may be in excess of 34,000 kDa^39^. Also LMW-GS are proposed to contain intramolecular disulphide bonds at their C-terminal domain homologous to the α/β and γ-gliadins^36^. The exact structure and composition of gluten polymers are not fully elucidated but partial reducing experiments allowed to infer a hierarchical structural organization^40^, so the accessibility of mTG to the reactive locations could be increased by reduction of the intermolecular disulphide bonds in glutenin macropolymer and also the intramolecular bonds present in α/β and γ-gliadins and LMW-GS. In order to test our hypothesis, transamidation reaction was performed under reducing conditions using 20 mM glutathione (GSH), using only *n*-butylamine as amine nucleophile as these flours showed good rheological properties. It was tested if lower concentrations of *n*-butylamine, under the reducing conditions would be able to hamper the excessive cross-linking of gluten proteins (Fig. 4a,b), but again only the use of 50-fold excess hampered an excessive cross-linking of gluten proteins (Wheat flour FRB-50X and gluten GRB-50X).

The use of reducing conditions for transamidation of glutamine residues resulted in a significant change in the RP-HPLC profile of gliadins (Fig. 4c,d). Additionally, also a significant change in glutenin profile was observed in the flour and gluten treated with 50-fold excess of *n*-butylamine under reducing conditions in relation to the mTG cross-linking action (Fig. 4e,f). This change in the glutenin RP-HPLC profile allows us to confirm, as hypothesized, that reduction conditions made glutenin proteins more accessible to the mTG allowing their modification, accessibility which was limited under non-reducing conditions. The increased retention time of gliadins for the FRB-50X and GRB-50X (Fig. 4c,d) as compared to FB-50X and GB-50X (Fig. 1c,d) also shows that under reducing conditions the extent of gliadin modification increases. These results are confirmed by the 7-fold increase in the γ-glutamyl-*n*-butylamine levels in gluten proteins under reducing conditions when compared to non-reducing conditions (Fig. 3a,b). Hence, using reducing conditions besides increasing the accessibility of glutenins held in gluten macropolymer^40^, conformational changes would also occur in the individual glutenins and gliadins by reducing the intramolecular disulphide bonds^23^. Also the amino acid analysis (Fig. 3a,c,f) shows a significant decrease in the content of glutamine residues in FRB-50X and GRB-50X in comparison to control flour (27%) and gluten (43%). There was no significant change in the lysine and glutamic acids levels for the FRB-50X and GRB-50X when compared to the unmodified flour and gluten, respectively (Fig. 3d,g,e,h).

### Improved gluten chemical modification decreases further the amount of toxic epitopes

Treatment of wheat flour with mTG without amine nucleophile under reducing conditions resulted also in a significant decrease, on average 68%, in the CD toxic epitope levels (Fig. 3i). But contrarily to what was observed for the non-reducing conditions (Fig. 2g), the treatment of wheat flour with a 50-fold *n*-butylamine molar excess under reducing conditions resulted in a significant decrease in the CD toxic epitopes of 94% (Fig. 3i), a significantly higher decrease than that observed for FK-C_2_H_5_-5X and FB-5X (Fig. 2g). The levels of toxic epitopes in the FRB-50X were also significantly lower than that found in the treatment with mTG alone, with an average decrease in reactivity of 80% (Fig. 3i). The same trend was observed when the gluten was treated under reducing conditions, giving a 94% significant decrease in toxic epitopes for the GRB-50X in comparison to the unmodified gluten (Fig. 3i). Also, a significant decrease of 81% in toxic epitopes in comparison to GRmTG was observed (Fig. 3i). The higher decrease in the toxic epitopes levels observed in the FRB-50X and GRB-50X when compared to FR, GR, FRmTG and GRmTG is not due to a different yield of 60% (v/v) ethanol soluble peptides resulting from the peptic-tryptic digestion (mass yield of 60% ethanol soluble peptides: FRB-50X–20.8%; FR–16.1%; FRmTG–17.0%; GRB-50X–116.4%; GR–110.6%; GRmTG–115.2%).

### Proteomics characterization

**Figure 5:**
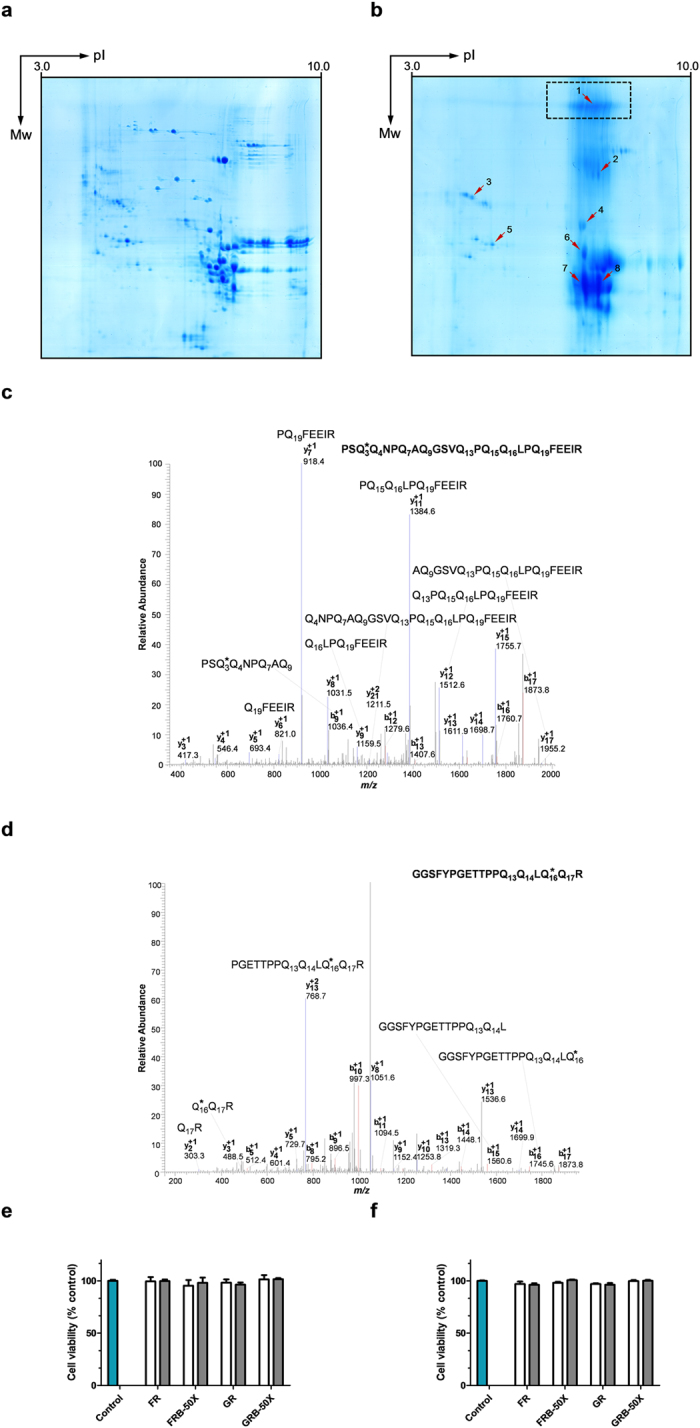
Proteomics and cytotoxicity analysis of *n*-butylamine bioconjugated gluten. Two-dimensional electrophoresis pattern (IEF × SDS-PAGE) of the gluten, original (**a**) and derivatised with *n*-butylamine (**b**) as amine nucleophile under reducing conditions. The arrowheads point to the excised and identified protein spots (please see Table 1, for spot identification). The dashed rectangle indicates protein aggregates. Tandem mass (MS/MS) spectra of (**c**) α-/β-gliadin derived tryptic digest peptide [precursor ion at *m/z* (+2) = 1396.2] from spot 7/8, excised from 2-DE gel of GRB 50X; (**d**) HMW-GS derived tryptic digest peptide [precursor ion at *m/z* (+2) = 1024.5] from spot 1, excised from 2-DE gel of GRB 50X. * indicates the modified glutamine residue. Alamar Blue (AB) assay for cell viability quantification using Caco-2 cells, after 24 h (**e**) or 48 h (**f**) treatment with different concentrations of peptic-tryptic digests of wheat flour and gluten, original and derivatised with *n*-butylamine, as amine nucleophile, under reducing conditions. For sample nomenclature please consult Fig. 6. A total of n = 3 different experiments (each with four replicates), from different cell passages. Error bars represent the standard deviation Empty bars (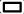) denote 0.5 mg/mL; and filled bars (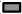) denote 1.0 mg/mL.

GR proteins were distinctly separated and most formed a single spot on the 2-D electrophoresis (2-DE) gel (Fig. 5a; Supplementary Fig. 1). There was a significant change in the 2-DE profile of the gluten proteins modified with *n*-butylamine (Fig. 5b) when compared to the untreated gluten (Fig. 5a), with the disappearance of some protein spots and appearance of protein aggregates (Fig. 5b, dashed rectangle). Proteins lacking in the GRB-50× 2-DE profile may be due to cross-linking between proteins, a polymeric curtain, absent in the GR control was noticed in the high molecular weight region of the GRB-50X. These high molecular weight proteins, probably retained by the stacking gel, hence not detected in the SDS-PAGE, are here revealed in the 2-DE performed without stacking gel^34^. In order to have a deeper insight into the modification of the GRB-50X proteins, the marked spots were analysed by nano-LC-ESI-MS/MS after in-gel tryptic digestion (Table 1). Indeed the spot identified in that changed area 1 corresponds to HMW-GS together with the presence of α/β-gliadin, α-amylase inhibitor and α-amylase/trypsin inhibitor with molecular weight much lower than the actual spot position. In similar way, spot 2 presents one HMW-GS and a γ-gliadin. Inversely the protein spots 3 and 5 present no modification in the glutamine residues and appear as clear distinct spots. With respect to some of the proteins (glutenin, α-amylase/trypsin inhibitor, α-amylase inhibitor, α/β-gliadin and γ-gliadin), it was possible to identify peptides with the same sequence but carrying the modification located at different glutamine residues within the sequence. This indicates sample heterogeneity occurring during the *n*-butylamine adduction to glutamine residues. No multi-modified peptides were identified in the spot samples. Furthermore, it should be noted the modification of the glutamine residues present in the PSQQ sequence of some peptides derived from GRB-50X proteins, namely α/β-gliadins (Table 1; Spots 1, 7 and 8), which is one of the most common sequences found to be active in celiac disease^41^. As an example of glutamine modification with *n*-butylamine of α-/β-gliadins and HMW-GS, Fig. 5c,d show the MS/MS spectra of peptides released after tryptic digestion of spots 7/8 and 1, respectively. Figure 5c shows the MS/MS fragmentation pattern of a precursor ion at *m/z*(+2) 1396.2 corresponding to the PSQ*QNPQAQGSVQPQQLPQFEEIR. Inspection of the resulting fragment ions belonging to the *b* and *y* ion series enables the identification of the modified Q residue at position 3. Specifically, taken together the presence of *b*9(+1) ion at *m/z* 1036.4 and *y*21(+2) at *m/z* 1211.5 strongly supports the modification at this position. A similar approach, based on the observed *b*n and *y*n ion series, was performed for confirming the other modification positions of this same peptide observed in Table 1 (Supplementary Fig. 2 to 6). Figure 5d shows the MS/MS fragmentation pattern of the precursor ion at *m/z*(+2) 1024.5 corresponding to the peptide sequence GGSFYPGETTPPQQLQ*QR. Also in this case the *y*n and *b*n ion series present strong evidence for the modification of the Q residue at position 16. Taken together the presence of *b*16(+1) ion at *m/z* 1745.6, *y*2(+1) ion at *m/z* 303.3 and *y*3(+1) at *m/z* 488.5 strongly supports the modification at this position (Supplementary Fig. 7 for the MS/MS fragmentation of unmodified peptide).

### Cytotoxicity evaluation

No free *n*-butylamine was detected by GC-MS showing that *n*-butylamine was efficiently removed by dialysis, being less than 5 ppm as recommended by National Institute for Occupational Safety and Health (NIOSH). Wheat flour and gluten derivatised with *n*-butylamine didn’t induce any cytotoxic effect in Caco-2 cells at the two assayed concentrations and at the two exposure times (Fig. 5e,f), in comparison to the untreated cells (control). Also, as *n*-butylamine had been shown to exert a high toxicity on Caco-2 cells, the absence of cell toxicity indicates that there were no toxic substances on the samples^42^.

### Gluten function is improved after modification

FR presented a resistance to extension not significantly different to the non-reduced unmodified flour (F), although the extension was significantly decreased. The fact that FR presented a similar R_max_ indicates that probably during dialysis, lyophilisation and storing during 4 months there was a reoxidation of the disulphide bonds that were broken by addition of GSH^43^,^44^. The loss in dough extensibility probably results from a protein reoxidation conferring a gluten structure different from the gluten present in the original flour, or possibly due to the intermolecular cross-linking of gliadins to the gluten macropolymer during the reoxidation process^44^ reducing extensibility. Treatment of wheat flour with mTG under reducing conditions (FRmTG) decreased significantly the force and the dough extensibility in comparison to unmodified reduced flour (FR), and was even lower than that obtained for the FmTG. The resistance to extension of the FRB-50X was significantly higher than that of the reduced unmodified flour and was also higher than that of the non-reduced unmodified flour, although presenting a lower resistance to extension when compared to FB-50X. Also the extensibility of the FRB-50X was significantly higher than that of FR and was not significantly lower than that of the non-reduced unmodified flour (F). The lower R_max_ of FRB-50X in relation to FB-50X can be due to a different arrangement of glutenins in the reoxidised flour as observed for the unmodified flour. Improvement of dough resistance to extension may be due to the higher hydrophobicity of gliadins and glutenins that can improve their association during gluten development^21^,^37^,^45^, although further analysis are required to make clearer the changes occurring in the gluten network.

## Discussion

Results obtained in this work show that the decrease in CD toxic epitopes in wheat flour and gluten treated under non-reducing conditions in the presence of amine nucleophiles is mainly due to the cross-linking activity of mTG and not to the transamidation of the glutamine residues with the amine nucleophile. This is explained by a limited accessibility of the enzyme to the reactive locations in glutenins and gliadins, and also due to the competition of albumins and globulins for the transamidation reaction in the case of flour^34^,^35^. Moreover, our results emphasize the need to solve such a complex structure as gluten to obtain an efficient chemical modification of toxic epitopes by mTG catalysed transamidation with amine nucleophiles, as for example many toxic epitopes are located in areas known to have conformations stabilized by disulphide bonds^46^,^47^, thereby hindering the enzyme activity.

Reaction extension is increased at least by 7-fold when gluten proteins are reduced prior to mTG catalysed transamidation, resulting in a significant decrease in toxic epitopes levels (94%) even when compared to wheat flour and gluten treated with mTG alone (80%).

The use of high molar excess of amine nucleophile is needed to decrease the cross-linking of gluten proteins by mTG previously to kneading in order to maintain good rheological properties of the resulting dough. Also flours modified with *n*-butylamine presented important advantages as the resulting proteins were more hydrophobic increasing dough’s rheological properties in comparison to the unmodified flour.

The simple, easily scale-up and efficient reaction developed in this work opens the possibility of tailoring gluten, and not only gliadins, by enzymatic modification with appropriate amine nucleophiles for producing flours with low levels of toxic epitopes while still taking advantage of the unique viscoelastic properties of wheat gluten. Nevertheless, demonstrating gluten safety is a complex task. The toxicity of gluten in CD arises from an immunoresponse involving both innate and adaptive systems, and immunoresponse varies widely between celiac patients^1^. At present, no model is available to replicate the CD immunoresponse, although rhesus macaques were recently proposed^48^. Thus, the demonstration of gluten toxicity depends on *in vivo* challenge studies^49^. Nevertheless, a randomized, single-blind, clinical study using bread made from wheat flour detoxified by transamidation of glutamine residues with lysine methyl ester showed that transamidated gluten reduced the number of clinical relapses in challenged patients with no changes of baseline values for serological/mucosal CD markers and an unaltered kidney function^12^.

As the chemical modification occurs specifically at glutamine residues of proteins and as glutamine residues are also present in the CD toxic epitopes, and mTG has a broad protein specificity^25^, this procedure can be potentially applied to other cereal products like rye and barley. Considering the serious nutritional and food restrictions imposed to celiac patients these findings provide new insight and a firm basis for the potential development of nutritional hypoallergenic products safe for celiac patients based on wheat gluten.

## Methods

### Transamidation of wheat flour and gluten

A commercial wheat flour (type 65, 7.8% protein content) without technological additives was used (“Espiga”, Fábricas Lusitana, Lisbon, Portugal). The gluten was obtained by aqueous washing of dough produced from wheat flour with the addition of 1 mol/L NaCl^33^.

**Figure 6:**
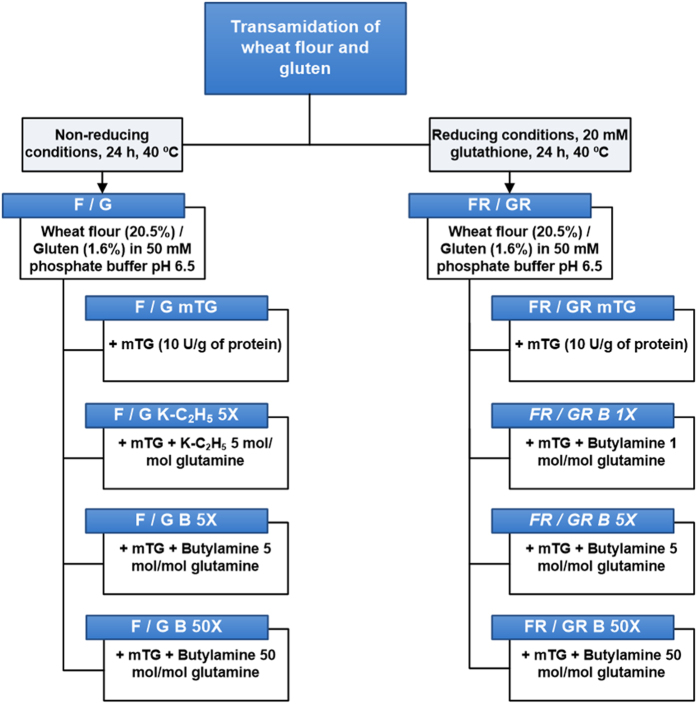
Reaction conditions of transamidation of wheat flour and gluten. The nomenclature and reaction conditions of the derivatised and control products. “F” denotes wheat flour and “G” denotes gluten. “R” denotes reducing conditions, “mTG” microbial transglutaminase, “B” *n-*butylamine and “K-C_2_H_5_” L-lysine ethyl ester. “1X”, “5X” and “50X” denote the different concentrations of amine nucleophile used.

Transamidation of wheat flour and gluten was performed in non-reducing conditions and in reducing conditions (Fig. 6). In non-reducing conditions, 5 mol L-lysine ethyl ester (Sigma-Aldrich, St. Louis, MO, USA)/mol of glutamine or 5 mol *n*-butylamine (Sigma-Aldrich, St. Louis, MO, USA)/mol of glutamine or 50 mol *n*-butylamine/mol of glutamine were solubilised in 50 mmol/L phosphate buffer pH 6.5 and pH was re-adjusted to 6.5, previously to wheat flour (20.5% w/v) and gluten (1.6% w/v) addition. Glutamine content was estimated taking an average value of 35% (w/w) in relation to the protein content^19^. After addition of the wheat flour or gluten, 10 U of microbial transglutaminase (mTG; EC 2.3.2.13) (ACTIVA®WM, Ajinomoto Foods, Hamburg, Germany) per gram of protein were added and the mixture was incubated under stirring for 24 h at 40 °C. At the end of this period, wheat flour and gluten were dialyzed (Molecular weight cut-off 12–14 kDa) against water (8 water renewals) under stirring at 4 °C and freeze dried yielding the flours FK-C2H5 5X, FB-5X and FB-50X, and the glutens GK-C2H5 5X, GB-5X and GB-50X (Fig. 6). Control samples were prepared using the same experimental conditions without mTG and the amine- nucleophile (flour F and gluten G), with mTG without the amine nucleophiles (flour FmTG and gluten GmTG) and without mTG with the amine nucleophiles. In the reducing conditions, the wheat flour and gluten were prepared using the same protocol, but with addition of 20 mmol/L glutathione (Sigma-Aldrich, St. Louis, MO, USA) before mTG addition and using only *n*-butylamine as amine nucleophile at three different levels (1 mol/mol glutamine, 5 mol/mol glutamine and 50 mol/mol glutamine) yielding the flours FRB-1X, FRB-5X and FRB-50X, and the glutens GRB-1X, GRB-5X and GRB-50X (Fig. 6). Control samples were prepared using the same experimental conditions without mTG and without the amine- nucleophiles (flour FR and gluten GR), with mTG and without the amine nucleophiles (flour FRmTG and gluten GRmTG), and without mTG with the amine- nucleophiles.

Transglutaminase activity of the commercial preparation (mTG units) were determined according to Folk and Chung^50^. One unit of enzyme is defined as the amount that catalyses formation of 1 μmol of the peptide derivative of γ-glutamylhydroxylamine per minute. Total protein content of commercial preparation was determined by the Biuret method. Specific activity is given as the units per g of protein (92.3 U/g).

### Electrophoresis

Gluten proteins, gliadins and glutenins, were extracted according to the sequential method of Singh *et al.*^51^. Gliadins were extracted with 50% (v/v) aqueous 1-Propanol. The supernatant was dried at 60 °C and the residue was dissolved in sample buffer. High molecular weight-glutenin subunits (HMW-GS) present in the pellet were reduced and alkylated in a 50% (v/v) 1-Propanol solution with 1% (w/v) dithiothreitol (DTT) and 2.5% (w/v) iodoacetamide, respectively. Gliadin and glutenin subunits were separated in a resolving gel using 10% and 12% T; 1.3% and 0.9% C, respectively. The gels were stained with Coomassie Blue R-250 for 24 h then washed in water overnight. Coomassie-stained gels were scanned with a flatbed scanner (Umax PowerLook 1100, Fremont, CA, USA).

Two-dimensional electrophoresis (2-DE) was carried out as previously described^52^. Briefly, total proteins were extracted with a solution containing 4% (w/v) CHAPS, 7 mol/L urea, 2 mol/L thiourea, 1% (v/v) immobilized pH gradient (IPG) buffer, 20 mmol/L DTT in milliQ ultrapure water. The flour plus the extraction solution were vortexed, sonicated and centrifuged. The rehydration of strips for isoelectric focusing (IEF, pH 3–10) was carried out using the rehydration solution consisting of extraction solution with bromophenol blue. IEF of 300 μg of protein was performed for a total of 60,000 Vh (linear gradient of 500 V for 2 h, linear gradient of 1,000 V for 3 h, linear gradient of 3,000 V for 3 h, linear gradient of 7,000 V for 3 h and finally 7,000 V for 5 h 10 min, making 16 h 10 min in total) on an Ettan™ IPGPhor II™ system (Amersham Biosciences, Uppsala, Sweden). Focused IPG strips were equilibrated twice for 15 min each in equilibration buffer [(6 mol/L urea, 30% (w/v) glycerol, 2% (w/v) sodium dodecyl sulphate (SDS) in 0.05 mol/L Tris–HCl buffer pH 8.8)]. In the first equilibration step, 1% DTT was added to the original equilibration buffer, and 4% iodoacetamide to the second step. Bromophenol blue was also added to both solutions. The equilibrated IPG strips were gently rinsed with sodium dodecyl sulphate (SDS) electrophoresis buffer, blotted to remove excessive buffer, and then applied to SDS-polyacrylamide gels (T = 12.52%, C = 0.97%). After sodium dodecyl sulphate-polyacrylamide gel electrophoresis (SDS-PAGE), the 2-D gels were fixed in 40% (v/v) methanol/10% (v/v) acetic acid solution for 1 h and then stained overnight in Coomassie Brilliant Blue G-250. Excess stain was removed by rinsing the gels with 40% (v/v) methanol solution. Coomassie-stained gels were scanned with a flatbed scanner (Umax PowerLook 1100; Fremont, CA, USA) and the digitized images were analysed using Lab Scanner Image Master 5.0 software (Amersham Biosciences; GE Healthcare) and Progenesis SameSpots v4.5 (Non-linear Dynamics Limited, Newcastle, UK). Protein patterns were the result of triplicate protein extractions and three 2-DE replicates. The reference gels are shown. Protein concentration was assayed using the 2D Quant kit (GE Healthcare, Buckinghamshire, UK) following the manufacturer’s instructions.

### RP-HPLC

Gliadins and glutenins were separated according to hydrophobicity by reversed phase-high performance liquid chromatography (RP-HPLC) conducted according to Wieser *et al.*^53^ and the extraction was carried out as described above and based on Singh *et al.*^51^. Briefly, gliadins are solubilized in 50% aqueous 1-propanol and the glutenins present in the pellet are solubilized in 50% aqueous 1-propanol with a reducing agent, dithiothreitol (1%), and alkylated with iodoacetamide (2.5%). For HPLC analysis, a RP-C8 column was used (25 cm, 4.5 mm i.d., 5 μm, Macherey-Nagel, Germany) maintained at 50 °C during the separation process, and an injection volume of 50 μL for glutenins and 100 μL for gliadins was used. A gradient elution was performed; eluent A consisting of 0.1% (v/v) aqueous trifluoroacetic acid and eluent B consisting of acetonitrile and trifluoroacetic acid (99.9/0.1%, v/v), with following elution program: 0 min 28% B, 30 min 56% B, flow rate of 1 mL/min. Detection was made by ultraviolet (UV) absorbance at 210 nm. After each analysis the column was cleaned by using 90% B for 5 minutes and equilibrated to 28% B over 10 min.

### Amino acid and γ-glutamyl-*n*-butylamine analysis by GC-MS

For amino acid analysis and γ-glutamyl-*n*-butylamine, the previously obtained peptic-tryptic digests (10 mg) were solubilized in 2 mL of 50 mmol/L phosphate buffer pH 7.0, containing 0.05% (w/v) sodium azide and 1% (w/w) Pronase (Sigma-Aldrich, Steinheim, Germany) and incubated at 37 °C for 24 h, procedure adapted from Marzilli *et al.*^54^. After this time, it was added 10 μg of Prolidase (Sigma-Aldrich, Steinheim, Germany) per sample and incubated at 37 °C for 2 h. The amino acids obtained after enzymatic hydrolysis were subsequently derivatised according to the method described by Qiu *et al.*^55^ and analysed by gas chromatography–mass spectrometry (GC-MS). Briefly, to 600 μL of amino acid solution, 400 μL of absolute ethanol and 100 μL of the internal standard, L-norleucine (0.1 mg/mL), were added. Then, 100 μL of pyridine and 50 μL of ethyl chloroformate were added. The mixture was sonicated for 60 s at 20 °C in an ultrasonic bath (Bandelin Sonorex RK 106 S, Germany). After this period, 300 μL of chloroform were added followed by addition of 100  μL of 7 mol/L NaOH and 50 μL of ethyl chloroformate. The phases were mixed by vortex for 30 sec and phase separation was performed by centrifugation for 3 min at 3000 rpm (Hettich EBA 8 S, Germany). The aqueous phase was removed and the organic phase was dried by adding 100 mg of anhydrous sodium sulphate. The organic phase was analysed by GC-MS (ThermoFinningam) using a non-polar column DB5-Inferno, 30 m, 0.25 cm internal diameter and 0.25 μm stationary phase, by injection of 1 μL in splitless mode (splitless time of 0.75 min) at a temperature of 260 °C. The initial column temperature was 80 °C, maintained for 2 min and the temperature increased to 140 °C at 10 °C/min and increased to 240 °C at a rate of 4 °C/min, and increased again to 280 °C at 10 °C/min and maintained at 280 °C for 3 min. The carrier gas (helium) was maintained at a constant flow rate of 1 mL/min. The transfer line temperature was 280 °C and the temperature of ionization source was 220 °C. Mass spectra were acquired in the full-scan mode (45–550 *m/z*) after ionization by electron impact with 70 eV electrons. The same ethyl chloroformate derivatization and GC-MS analysis protocol were used for *n*-butylamine analysis. Quantification of glutamine, lysine, glutamic acid, phenylalanine and *n*-butylamine was performed by the internal standard method. The amino acid results were normalized to the phenylalanine content of the hydrolysate in order to account for variations in the total protein content of samples. Analysis was performed in triplicate.

### Identification of γ-glutamyl-*n*-butylamine by CI-MS and CI-MS^2^

In the amino acids analysis chromatograms of wheat flour and gluten modified with *n*-butylamine an abundant peak with a retention time of 31.54 min was observed (Fig. 3a). The identification of this peak as γ-glutamyl-*n*-butylamine was confirmed by chemical ionization mass spectrometry using methane as the reagent gas (Supplementary Fig. 8b) and CI-MS^2^ of the pseudo-molecular ion (M + H)+ at *m/z* 303 (Supplementary Fig. 8c). For CI-MS, chromatographic separations was performed as previously described, the transfer line temperature was 280 °C and the temperature of ionization source was 100 °C and methane was used as ionization gas at a flow of 2 mL/min. Mass spectra were acquired in the full-scan mode (45–550 *m/z*) after ionization by electron impact with 70 eV electrons. For MS^2^ experiments, the ion at m/z 303 was selected with a width of 1.0 during 12 ms, and a voltage of 1 and medium energy was used for fragmentation, and mass spectra were acquired in the full-scan mode (101–303 *m/z*). Semi-quantification of γ-glutamyl-*n*-butylamine was performed by normalization of the area to that of phenylalanine of the hydrolysate in order to account for variations in the total protein content of samples. Analysis was performed in triplicate.

### *In vitro* wheat flour and gluten digestion and R5 Competitive ELISA immunoassay

For quantification of celiac patients’ toxic epitopes present in wheat flour and gluten, original and derivatised with L-lysine ethyl ester or *n*-butylamine, the commercial product RIDASCREEN® Gliadin competitive (R-Biopharm AG, Darmstadt, Germany) was used. This product is based on an enzyme-linked immunosorbent assay (ELISA) with competitive format and the R5 monoclonal antibody, recognizing as core sequences the toxic QQPFP, QQQFP, LQPFP, QLPFP, QLPYP, among others that occur repeatedly in the proteins of gluten. The official standard method for gluten determination according to the Codex Alimentarius is an ELISA which uses the R5 antibody; this requirement is fulfilled by the RIDASCREEN® Gliadin competitive. The format of this competitive assay has the advantage of detecting individual peptide fragments compared to the sandwich ELISA format. The detection limit is 1.36 ppm of gliadin and the quantification limit is 5 ppm of gliadin. In order to avoid potential artefacts due to the possibility of changing the solubility properties of the gliadins and glutenins, either due to the structural changes imposed when modified with the amine- nucleophile or due to cross-linking, the flours and glutens were submitted to a peptic-tryptic digestion process in order to release small peptides soluble in 60% (v/v) ethanol, the extraction solvent used in the R5 kit.

All instructions of RIDASCREEN® Gliadin competitive product were strictly followed and the preparation of the material was done as described by Gessendorfer *et al.*^56^. Briefly, wheat flour and gluten, original and derivatised, were dispersed in distilled water at a ratio of 5% (w/v) and the pH was adjusted to 1.8 using a solution of 1 mol/L HCl. Thereafter, 2.5 mg of pepsin (Sigma-Aldrich, Steinheim, Germany) was added to react for four hours under stirring at 37 °C. Subsequently the pH was adjusted to 7.8 with NaOH 1 mol/L and 2.5 mg of trypsin (Sigma-Aldrich, Steinheim, Germany) was added to the mixture to react for other four hours under stirring at 37 °C. Finally, the pH was adjusted to 4.5 with a solution of 1 mol/L HCl and peptic-tryptic digests were centrifuged at 4000 x*g* for twenty minutes at room temperature. The supernatant was decanted and freeze dried. The dry residue obtained, i.e., peptic-tryptic digests (PT) were solubilized in 60% (v/v) aqueous ethanol as described in the protocol RIDASCREEN® Gliadin competitive for subsequent analysis. Several dilutions changing the standard curve were performed for better quantification of the different samples. *n* = 2 different experiments (each with four replicates).

### Micro-extension tests

In order to study the effect of the chemical modification of wheat flour with mTG alone and in the presence of amine nucleophiles, the rheological properties of the corresponding dough were studied. Gluten and dough are unique from the point of material science due to their complex behaviours. Small and large deformation tests have been used for measuring dough rheological properties, but large deformation tests are more suitable for evaluating their application as food as the measures can be related with its eating quality^57^. A commonly used large deformation test of dough is extension. It has been shown that resistance to extension of dough is related with its protein quality, along with the presence of specific HMW-GS and high proportions of unextractable protein^57^, and extension allows a reliable prediction of loaf volume^58^. Micro-extension tests on dough were performed as previously described^58^. Briefly, after a resting time of 20 min at 30 °C under a water saturated atmosphere, dough [(flour to water ratio of approximately 1.8 (w/v)] was brushed with paraffin oil, to avoid sample adhesion, and pressed into a Teflon mould pre-warmed to 30 °C, allowed to stand for a further 40 min at 30 °C under a water saturated atmosphere, and then were measured. The Teflon mould and the measuring instrument for dough were the SMS/Kieffer Dough and Gluten Extensibility Rig with the Texture Analyser TA-XT2 (Stable Micro Systems). F+mTG designates the dough prepared using control flour and mTG added (10 U/g of protein) during kneading. Analysis was performed on duplicate.

### Mass spectrometry analysis

2-DE gel pieces were excised and prepared for tryptic digestion. Briefly and according to Wilm *et al.*^59^, gel pieces were washed with 50–100 μL of a mix (1:1) of acetonitrile (ACN) and ammonium bicarbonate (NH_4_HCO_3_) (25 mmol/L, pH 8.0). Two or three washes were repeated to remove as much stain as possible and then discarded. Gel pieces were then dried with 20–50 μL of ACN for 10 min, ACN was discarded, and gel pieces were dried under vacuum. Sequencing grade modified trypsin (AbSCIEX) (25 μL of 12.5 ng/μL in 25 mmol/L NH_4_HCO_3_) was added. Extra buffer was added after 1 h, if necessary to maintain gel pieces hydrated during overnight incubation at 37 °C. Following digestion, 25 μL of 10% (v/v) formic acid was added as an extraction solution. After 30 min, supernatant was reserved and the acidic extraction, with a mix (1:1) of ACN and 10% formic acid, was repeated twice. For each gel piece, all supernatants were pooled and each resulting peptide mixture was then dried under vacuum. For nano-liquid chromatography-electrospray-ion trap-tandem mass spectrometry (Nano-LC-ESI-IonTrap-MS/MS) analysis and protein identification, the dried tryptic peptides were dissolved in 20 μL of mobile phase A [0.1% formic acid (FA), 5% ACN] and then separated using an Ultimate 3000 (Dionex, Sunnyvale, CA, USA). Peptides separation was carried out in a 150 mm × 75 μm Pepmap100 capillary analytical C-18 column with 3 μm particle size (Dionex, LC Packings) at a flow rate of 300 nL/min. The gradient started at 10 min and ramped to 50% Buffer B (85% ACN, 0.1% FA) over a period of 45 min. The chromatographic separation was monitored at 214 nm using a UV detector (Dionex/LC Packings) equipped with a 3 nL flow cell. Peptides eluting from the capillary tip were introduced into the a linear ion trap mass spectrometer (LXQ, Thermo Finnigan, San Jose, CA, USA) equipped with a nanoelectrospray source operating with a capillary voltage of 1.8 kV and at a temperature of 200 °C, and performing a full scan and precursor selection in the range of *m/z* 300−1700. MS/MS spectra were recorded using dynamic exclusion of previously analysed precursors for 45 sec with a repeat of 1 and a repeat duration of 2. MS/MS data were evaluated using the TuboSequest algorithm of the Bioworks 3.1 software (Thermo Electron Corporation) and searches were performed against the SwissProt database for *Triticum*. Data on Sequest was filtered using Xcorr score thresholds of ≥2 for 1 + peptides, Xcorr ≥ 2.5 for 2 + peptides, and Xcorr ≥ 3 for 3 + peptides with Rsp ≤ 5 and Sp ≥ 350. All peptide samples were analysed two separate times. All tandem mass (MS/MS) spectra were manually analysed utilizing Data Explorer software TM v4 (Applied Biosystems). Modification assignment was considered if a given spectrum contained peaks to confirm both the peptide’s identity and modification. The modifications considered during spectra interpretation included *n*-butylamine transamidation of glutamine residues. In order to cross‐validate our manual interpretation and minimize the occurrence of false positive results, the experimental data was also processed with the Protein Prospector software. This approach permitted the identification and assignment of a mass shift to a given amino acid residue.

### Cytotoxicity assay

Caco-2, a human colorectal adenocarcinoma cell line (Cell Line Services, AG, Eppelheim, Germany) was maintained in culture media consisting of Dulbecco’s modified Eagle’s medium (DMEM) supplemented with 10% (v/v) foetal bovine serum (FBS), 1 mmol/L L-glutamine and antibiotics (100 U/mL penicillin and 100 μg/mL streptomycin) at 37 °C, 5% CO2/95% air environment and with controlled humidity (CO_2_ incubator, Binder).

For the cytotoxicity assay cells were detached from the culture flaks with trypsin, counted and seeded into 96-well microplates at a density of 5 × 10^4^ cells/mL (100 μL/well). After 24 h of culture (CO_2_ incubator), the culture media was removed, cells were washed and were exposed to PT digests solubilised in FBS-free culture media at given concentrations (0.5 and 1.0 mg/mL), each replicates for each concentration and exposure time. After 24 h or at 48 h of incubation with PT digests, the culture media was removed, cells were washed and Alamar Blue (Alfagene, Invitrogen, Portugal) solution [10% (v/v) in FBS-free culture medium] was added into each well (100 μL/well). Cell viability was quantified using the Alamar Blue (AB) Assay^60^, which consists of a water-soluble formazan dye that is non-toxic, diffuses into cells and allows the monitoring of metabolic activity of living cells. Innate metabolic activity results in dye conversion from its oxidized form (resazurin; blue) to the reduced form (resorufin; pink), which is accompanied by a colour change. The percentage of reduced AB can be considered proportional to the sample percentage of living cells. Absorbance at 570 (A570) and 620 (A620) nm were read, 5 h after AB addition, using a microplate reader (Multiskan EX, MTX Labsystems, USA). The percentage of AB reduction was calculated according to the manufacturer’s guidelines. Results are expressed as a percent of living cells as compared with untreated controls.

### Statistical Analysis

The results are expressed as mean ± standard deviation (SD). Differences among the different treatment groups were determined by one-way analysis of variance (ANOVA). Multiple comparisons of treatment means were made using the Tukey’s post-hoc test, and the criterion for significance was *p* < 0.05 (GraphPad Prism v6.03, GraphPad Software, La Jolla, California, USA).

## Additional Information

**How to cite this article**: Ribeiro, M. *et al.* Efficient chemo-enzymatic gluten detoxification: reducing toxic epitopes for celiac patients improving functional properties. *Sci. Rep.* **5**, 18041; doi: 10.1038/srep18041 (2015).

## Supplementary Material

Supplementary Information

## Figures and Tables

**Table 1:** Protein identification from nano-liquid chromatography-electrospray-ion trap-tandem mass spectrometry analysis.

Spotnumber	Protein ID	Accessionnumber	Genename	Mw(Da)	Peptide sequence	Start-Endsequence	P (pep)	z	Peptidemass (Da)
**1**	Glutenin, high molecular weight subunit PW212	P08489	*GLT4*	89119.8	K.GGSFYPGETTPPQQLQQR.I	82–99	1.73E-09	2	1990.9617
					K.GGSFYPGETTPPQQLQ*QR.I		1.54E-04	2	2048.0322
	Alpha/beta-gliadin	P02863	*GDA0*	32942.7	R.PSQQNPQAQGSVQPQQLPQFEEIR.N	233–256	2.35E-08	3	2734.3543
	Alpha-amylase inhibitor 0.19	P01085	*IAA1*	13328.4	R.LQ*CNGSQVPEAVLR.D	26–39	2.18E-06	2	1570.8496
					R.LQCNGSQ*VPEAVLR.D		3.27E-05	2	1570.8496
	Alpha-amylase/trypsin inhibitor CM3	P17314	*IAAC3*	18209.1	R.DYVLQQ*TCGTFTPGSK.L	45–60	4.69E-06	2	1801.8915
				R.DYVLQ*QTCGTFTPGSK.L		7.33E-04			
**2**	Glutenin, high molecular weight subunit DY10	P10387	*GLT0*	69586.7	K.GQQGYYPTSLQQPGQGQQGYYPTSLQHTGQR.Q	185–215	1.89E-13	3	3453.6207
	Gamma-gliadin	P08453	*GDB2*	37098.8	R.PFIQPSLQQQ*LNPCK.N	171–185	2.63E-05	2	1797.9806
					R.PFIQPSLQQ*QLNPCK.N		3.05E-04		
**3**	Beta-amylase	P93594	*AMYB*	56575.2	R.NIEYLTLGVDDQPLFHGR.T	129–146	8.54E-12	2	2087.0556
**4**	Alpha-amylase/trypsin inhibitor CM3	P17314	*IAAC3*	18209.1	R.DYVLQQ*TCGTFTPGSK.L	45–60	1.17E-05	2	1801.8915
					R.DYVLQ*QTCGTFTPGSK.L		1.42E-04		
					R.LLVAPGQ*CNLATIHNVR.Y	141–157	5.71E-05	3	1876.0711
	Alpha-amylase inhibitor 0.19	P01085	*IAA1*	13328.4	R.LQ*CNGSQVPEAVLR.D	26–39	8.06E-05	2	1570.8496
					R.LQCNGSQ*VPEAVLR.D		9.94E-04		1570.8496
**5**	Serpin-Z1A	Q41593	*SPZ1A*	43091.2	K.LVLANALYFK.G	172–181	5.14E-08	2	1151.6823
					K.LSAEPDFLER.H	261–270	5.30E-08	2	1176.5895
					K.AAEVTTQVNSWVEK.V	138–151	5.15E-07	2	1561.7857
					K.ISFGIEASDLLK.C	189–300	2.59E-06	2	1292.7096
					K.LSAEPDFLER.H	261–270	9.06E-05	1	1176.5895
**6**	Alpha-amylase inhibitor 0.19	P01085	*IAA1*	13328.4	R.LQ*CNGSQVPEAVLR.D	26–39	3.10E-06	2	1570.8496
					R.LQCNGSQ*VPEAVLR.D		5.12E-04		
	Gamma-gliadin	P08453	*GDB2*	37098.8	R.PFIQPSLQQQ*LNPCK.N	171–185	1.21E-05	2	1797.9806
					R.PFIQPSLQQ*QLNPCK.N		1.67E-04		
					R.APFASIVAGIGGQ	315–327	9.62E-04	1	1187.6419
**7**	Alpha/beta-gliadin	P02863	*GDA0*	32942.7	R.PSQQNPQAQGSVQPQQLPQFEEIR.N	233–256	1.67E-14	2	2734.3543
					R.PSQQNPQAQ*GSVQPQQLPQFEEIR.N		4.44E-07	2	2791.4248
					R.PSQQNPQ*AQGSVQPQQLPQFEEIR.N		4.17E-05	2	
					R.PSQQNPQAQ*GSVQPQQLPQFEEIR.N		2.31E-04	3	
					R.PSQQ*NPQAQGSVQPQQLPQFEEIR.N		3.46E-04	2	
					R.PSQ*QNPQAQGSVQPQQLPQFEEIR.N		3.46E-04	2	
					R.PSQQNPQAQGSVQ*PQQLPQFEEIR.N		3.84E-04	2	
	Gamma-gliadin	P08453	*GDB2*	37098.8	R.PFIQPSLQQQ*LNPCK.N	171–185	6.44E-05	2	1797.9806
					R.PFIQPSLQQQ*LNPCK.N		6.42E-04	3	
					R.PFIQPSLQQ*QLNPCK.N		6.64E-04	2	
**8**	Alpha/beta-gliadin	P02863	*GDA0*	32942.7	R.PSQQNPQAQGSVQPQQLPQFEEIR.N	233–256	3.07E-08	2	2734.3543
					R.PSQQNPQAQ*GSVQPQQLPQFEEIR.N		4.25E-07	2	2791.4248
					R.PSQQNPQ*AQGSVQPQQLPQFEEIR.N		5.04E-05		
					R.PSQ*QNPQAQGSVQPQQLPQFEEIR.N		4.35E-04		
					R.PSQQ*NPQAQGSVQPQQLPQFEEIR.N		4.35E-04		
	Gamma-gliadin	P08453	*GDB2*	37098.8	R.APFASIVAGIGGQ	315–327	3.49E-05	2	1187.6419
					R.PFIQPSLQQQ*LNPCK.N	171–185	5.53E-04	2	1797.9806

Q^*^ γ-glutamyl-*n*-butylamine.
