# Supplementary material for: Efficient chemo-enzymatic gluten detoxification: reducing toxic epitopes for celiac patients improving functional properties
Source: Sci Rep. 2015 Dec 22;5:18041. doi: 10.1038/srep18041 (PMC4686914; doi:10.1038/srep18041)
Supplement: Supplementary Information [file srep18041-s1.doc]

***Supplementary Information***

**Efficient chemo-enzymatic gluten detoxification: reducing toxic epitopes for celiac patients improving functional properties**

*Miguel Ribeiro ^1,2,3^, Fernando M. Nunes ^1,*^, Sofia Guedes ^4^, Pedro Domingues ^4^, Amélia M. Silva ^5^, Jose Maria Carrillo ^6^, Marta Rodriguez-Quijano ^6^, Gérard Branlard ^7^ and Gilberto Igrejas ^2,3^*

^1^ CQ-VR, Chemistry Research Centre, Chemistry Department, University of Trás-os-Montes and Alto Douro, 5000-801 Vila Real, Portugal;

^2^ Department of Genetics and Biotechnology, University of Trás-os-Montes and Alto Douro, 5000-801 Vila Real, Portugal;

^3^ Functional Genomics and Proteomics Unity, University of Trás-os-Montes and Alto Douro, 5000-801 Vila Real, Portugal;

^4^ Chemistry Department, University of Aveiro, 3810-193 Aveiro, Portugal;

^5^ Centre for the Research and Technology of Agro-Environmental and Biological Sciences, University of Trás-os-Montes and Alto Douro (CITAB-UTAD), 5000-801 Vila-Real, Portugal;

^6^ Unidad de Genética y Mejora de plantas Departamento de Biotecnología, E.T.S. Ingenieros Agrónomos Universidad Politécnica de Madrid, 28040 Madrid, España;

^7^ Institut National de la Recherche Agronomique GDEC/UBP, UMR 1095, 63100 Clermont-Ferrand, France.

***** To whom correspondence should be addressed

e-mail: fnunes@utad.pt

Phone: +351259350242

**Supplementary Figure 1. Two-dimensional electrophoresis pattern (IEF × SDS-PAGE) of the wheat flour proteins (FRB-50X)**. (**a**) unmodified proteins and (**b**) derivatized with *n*-butylamine. The dashed rectangle indicates protein aggregates.

**Supplementary Figure 2. MS/MS analysis of the non-modified α-/β-gliadin derived tryptic digest peptide.** Tandem mass (MS/MS) spectra of non-modified α-/β-gliadin derived tryptic digest peptide [precursor ion at m/z (+2) = 1396.2] from spot 7/8, excised from 2-DE gel of GRB-50X.

**Supplementary Figure 3. MS/MS analysis of the α-/β-gliadin derived tryptic digest peptide modified at position 13.** Tandem mass (MS/MS) spectra of α-/β-gliadin derived tryptic digest peptide [precursor ion at m/z (+2) = 1367.7] from spot 7/8, excised from 2-DE gel of GRB-50X. * indicates the modified glutamine residue.

**Supplementary Figure 4. MS/MS analysis of the α-/β-gliadin derived tryptic digest peptide modified at position 9.** Tandem mass (MS/MS) spectra of α-/β-gliadin derived tryptic digest peptide [precursor ion at m/z (+2) = 1367.7] from spot 7/8, excised from 2-DE gel of GRB-50X. * indicates the modified glutamine residue.

**Supplementary Figure 5. MS/MS analysis of the α-/β-gliadin derived tryptic digest peptide modified at position 7.** Tandem mass (MS/MS) spectra of α-/β-gliadin derived tryptic digest peptide [precursor ion at m/z (+2) = 1367.7] from spot 7/8, excised from 2-DE gel of GRB-50X. * indicates the modified glutamine residue.

**Supplementary Figure 6. MS/MS analysis of the α-/β-gliadin derived tryptic digest peptide modified at position 4.** Tandem mass (MS/MS) spectra of α-/β-gliadin derived tryptic digest peptide [precursor ion at m/z (+2) = 1367.7] from spot 7/8, excised from 2-DE gel of GRB-50X. * indicates the modified glutamine residue.

**Supplementary Figure 7. MS/MS analysis of the of non-modified HMW-GS derived tryptic digest peptide.** Tandem mass (MS/MS) spectra of non-modified HMW-GS derived tryptic digest peptide [precursor ion at m/z (+2) = 996.0] from spot 1, excised from 2-DE gel of GRB-50X.

| **a** | |
| --- | --- |
|  | |
| **b** | **c** |
|  |  |

**Supplementary Figure 8.** **Confirmation of the identity of γ-glutamyl-*n*-butylamine by CI-MS and CI-MS^2^**. **(a)** Fragmentation of γ-glutamyl-*n*-butylamine under EI (**Fig 4a**). **(b)** CI (CH4) mass spectra of γ-glutamyl-*n*-butylamine. **(c)** MS^2^ of ion *m/z* 303. The interpretation of the EI mass spectra of γ-glutamyl-*n*-butylamine and the assignment of various ions were based on the previous knowledge on fragmentation behaviour of the N-ethoxycarbonyl amino acid ethyl esters (ECEE’s)[^1^](#_ENREF_1). In EI spectra of γ-glutamyl-*n*-butylamine (**Fig. 4a**) the molecular ion peak at *m/z* 302 is absent, a common feature of the EI mass spectra of ECEE’s. For purpose of molecular weight confirmation, a CI (CH_4_) mass spectrum was acquired and is shown in **(b)**, were the ion at *m/z* 303 was assigned [M+H]^+^, being observed also the ions at [M+29]^+^ and [M+41]^+^, representing the adducts with C_2_H_5_^+^ and C_3_H_5_^+^, respectively, as is typical when methane is used as the chemical ionization reagent gas. In order to further confirm the ion *m/z* 303 as the [M+H]^+^ ion, a MS^2^ mass spectra was obtained and is shown in **(c)**. The EI spectrum in **Fig. 4a** shows the base peak at *m/z* 140 and the peak at the highest value of *m/z* 257. On **(a)** describes the fragmentation route observed for γ-glutamyl-*n*-butylamine. The peak at *m/z* 257 is formed by the loss of CH_3_CH_2_O^•^ resulting from the α-cleavage of the ECEE carboxylic ester group. The prevailing route is the loss of the a ^•^CO_2_Et that initiates a sequence reaction leading to the cyclization induced by functional group interaction between -CONH-, and the amino terminus in the EE+ species (*m/z* 229 = M – ^•^CO_2_Et) as described for ECEE glutamine fragmentation.

**Supplementary references**

1. Huang, Z.-H. et al. Characterization of N-ethoxycarbonyl ethyl esters of amino acids by mass spectrometry. *J. Chromatogr. A* **635**, 271-281 (1993).
